# Coincidence cloning recovery of *Brucella melitensis* RNA from goat tissues: advancing the in vivo analysis of pathogen gene expression in brucellosis

**DOI:** 10.1186/s12867-018-0111-x

**Published:** 2018-08-01

**Authors:** Paola M. Boggiatto, Daniel Fitzsimmons, Darrell O. Bayles, David Alt, Catherine E. Vrentas, Steven C. Olsen

**Affiliations:** Infectious Bacterial Diseases Research Unit, National Animal Disease Center, Agricultural Research Service, U.S. Department of Agriculture, Ames, IA 50010 USA

**Keywords:** Brucella, Brucellosis, *B. melitensis*, Coincidence cloning, Pathogen RNA, RNA-Seq

## Abstract

**Background:**

*Brucella melitensis* bacteria cause persistent, intracellular infections in small ruminants as well as in humans, leading to significant morbidity and economic loss worldwide. The majority of experiments on the transcriptional responses of *Brucella* to conditions inside the host have been performed following invasion of cultured mammalian cells, and do not address gene expression patterns during long-term infection.

**Results:**

Here, we examine the application of the previously developed coincidence cloning methodology to recover and characterize *B. melitensis* RNA from the supramammary lymph node of experimentally-infected goats. Using coincidence cloning, we successfully recovered *Brucella* RNA from supramammary lymph nodes of *B. melitensis*-infected goats at both short-term (4 weeks) and long-term (38 weeks) infection time points. Amplified nucleic acid levels were sufficient for analysis of *Brucella* gene expression patterns by RNA-sequencing, providing evidence of metabolic activity in both the short-term and the long-term samples. We developed a workflow for the use of sequence polymorphism analysis to confirm recovery of the inoculated strain in the recovered reads, and utilized clustering analysis to demonstrate a distinct transcriptional profile present in samples recovered in long-term infection. In this first look at *B. melitensis* gene expression patterns in vivo, the subset of *Brucella* genes that was highly upregulated in long-term as compared to short-term infection included genes linked to roles in murine infection, such as genes involved in proline utilization and signal transduction. Finally, we demonstrated the challenges of qPCR validation of samples with very low ratios of pathogen:host RNA, as is the case during in vivo brucellosis, and alternatively characterized intermediate products of the coincidence cloning reaction.

**Conclusions:**

Overall, this study provides the first example of recovery plus characterization of *B. melitensis* RNA from in vivo lymph node infection, and demonstrates that the coincidence cloning technique is a useful tool for characterizing in vivo transcriptional changes in *Brucella* species. Genes upregulated in long-term infection in this data set, including many genes not previously demonstrated to be virulence factors in mice or macrophage experiments, are candidates of future interest for potential roles in *Brucella* persistence in natural host systems.

**Electronic supplementary material:**

The online version of this article (10.1186/s12867-018-0111-x) contains supplementary material, which is available to authorized users.

## Background

*Brucella* are Gram-negative, facultative intracellular bacteria that cause brucellosis in animals and humans. Brucellosis is one of the most common bacterial zoonotic diseases worldwide with approximately 500,000 new infections annually [1]. *Brucella melitensis* infects primarily sheep and goats, resulting in abortion, decreased production, and infertility in infected animals. *B. melitensis* is also highly pathogenic to humans. Human brucellosis caused by *B. melitensis* has a high incidence in developing countries and is considered one of the seven neglected zoonoses by the World Health Organization (WHO) [2].

*Brucella* species have a complex pathophysiology, characterized by their ability to evade the host immune response. *Brucella* survive and replicate inside phagocytic cells of the immune system, primarily macrophages and dendritic cells, and modulate host cell function (reviewed in [3]). Phagocytic cells serve as an infection niche and also allow *Brucella* spp. to disseminate to other tissues, including the reticuloendothelial system and the reproductive tract of both males and females. *Brucella* spp. have evolved a variety of mechanisms for intracellular survival including inhibition of apoptosis of infected cells, remodeling of the phagocytic pathway, and modulation of host cell signaling inflammatory pathways [3–13]. These strategies enable long-term survival within infected cells and facilitate the establishment of chronic infection. Ultimately, the intracellular nature *of Brucella* spp. lends itself to the protracted and often clinically silent evolution of the disease.

The intracellular nature of *Brucella* not only limits its exposure to the host immune system, but also complicates the study of host–pathogen interactions in vivo, which requires an understanding of both the host’s response to infection and the pathogen’s response to the host. The intracellular environment poses a challenge for survival, as *Brucella* spp. face nutrient starvation, low pH, and low oxygen tension conditions [14–17]. While *Brucella* lack classical virulence factors (e.g., exotoxins, cytolysins, exoproteases) [18, 19], many other virulence genes have been characterized, encompassing multiple functional categories, with an overrepresentation of genes involved in intracellular trafficking and vesicular transport, transcription, cell wall and membrane biogenesis, nucleotide transport and metabolism, and cell motility (reviewed in [20–22]). While a few studies have been performed utilizing tagged mutant libraries of *Brucella* in order to identify genes necessary for survival in the mammalian host [23–26], many genes associated with virulence were identified from studies done in cell culture using macrophage infections, or in vitro using pure *Brucella* cultures under different growth conditions. These studies have provided insight into the genes important for *Brucella* pathogenicity in model systems, yet very little is known regarding the gene expression profiles of *Brucella* within an infected host. Recently, Rossetti et al. [27] characterized the transcriptional profile of *B. melitensis* in experimentally-infected jejuno-ileal segments of calves over a 4-h time period. However, no studies have yet characterized *Brucella* transcriptional profiles during long-term natural host infection.

The current affordability of whole-genome transcriptome studies provides the ability to characterize gene expression profiles across large numbers of samples. However, our ability to apply these techniques during in vivo infections is limited by the availability of pathogen RNA in host-derived tissue samples, especially in the case of chronic infections with low bacterial loads and low ratios of pathogen RNA relative to host RNA. In order to circumvent this problem of excess host RNA, several techniques have been developed, including differential lysis of eukaryotic vs. prokaryotic cells, subtractive hybridization to enrich bacterial transcripts, and hybridization-based selection of bacterial transcripts (summarized by [28]). For *Brucella*, Rossetti et al. [29] developed a methodology for bacterial enrichment that involves both selective reduction of host transcripts and selective amplification of *B. melitensis* open reading frames with a set of 89 primers. These primers were validated at pathogen:host RNA ratios of 1:12.5, which is a much larger ratio than that expected for our lymph node samples in chronic infection.

Recently, Azhikina et al. [28] developed a coincidence cloning-based methodology for whole pathogen transcriptome analysis, which allows for isolation of representative bacterial RNA from small quantities of infected tissues in the absence of additional bacterial genomic sequence information. Coincidence cloning involves hybridization of excess bacterial genomic DNA with cDNA derived from RNA extracted from infected tissues, with subsequent selective amplification of the prokaryotic fraction of the cDNA sample. Therefore, coincidence cloning is potentially applicable to samples with very low concentrations of bacteria per weight of host tissue (estimated at 0.04%, or a pathogen:host ratio of 1:2500, in the case of tuberculosis samples [30]). This methodology was previously utilized to characterize the transcriptome of *Mycobacterium tuberculosis* isolated from mouse lung tissues [28], and was able to identify upregulated genes in lung-derived samples from two different mouse strains [31].

Here, we examined whether coincidence cloning could be used to detect and characterize the transcriptome of *B. melitensis* strain 16M in the tissues of experimentally-infected goats. Application of the methodology to supramammary lymph node samples allowed for high-coverage RNA-sequencing analysis of samples from goats with short-term and long-term *Brucella* infections. We observed clustering of gene expression profiles for the long-term infection group, providing evidence that the method can be used for differential expression profiling. We also characterized some of the challenges related to the validation of results for low bacterial abundance systems. In sum, the data presented here indicate the utility of coincidence cloning in the study of host–pathogen interactions during *Brucella* spp. infection and provide the first characterization of the *Brucella* transcriptome post-dissemination in the natural host.

## Methods

### Bacterial cultures

*Brucella melitensis* strain 16M was obtained from the National Animal Disease Center (Ames, IA) culture collection. Frozen stock cultures used for experimental infection or reagent preparation were propagated on tryptose agar (Difco Laboratories, Detroit, MI) containing 5% bovine sera (TSA) for 72 h at 37 °C and 5% CO_2_. Bacteria were harvested via resuspension in phosphate-buffered saline (PBS), and bacterial concentrations were determined via measurement of the optical density at 600 nm with a spectrophotometer plus an OD_600_/CFU calibration curve. Final concentrations of live bacteria used for animal challenges were determined by serial dilution and standard plate counts on TSA. For use in serology assays, strain 16M bacteria were grown on TSA for 48 h at 37 °C and resuspended in PBS; bacterial concentrations were determined by standard plate counts. After incubation at 60 °C for 2 h, and confirmation of inactivation by microbiological culture, aliquots of the culture suspension were stored at − 80 °C until use. Culture manipulations were performed in a certified biosafety cabinet in a select agent-registered space, using biosafety level (BSL)-3-level precautions.

### Experimental challenge of goats

Seven female goats of approximately 1–3 years of age were obtained from brucellosis-free herds. The group contained Toggenburg, Alpine, and Saanen breeds. After onsite acclimation for 2 weeks, animals were moved into an agricultural biosafety level (AgBSL)-3 facility at the National Animal Disease Center (NADC) in Ames, Iowa, and allowed to acclimate for an additional 2 weeks prior to intraconjunctival challenge with 10^7^ *B. melitensis* strain 16M. Animals were divided into short-term (4 weeks; n = 3; all pregnant) and long-term (38 weeks; n = 4; one pregnant, three non-pregnant) infection groups. All were maintained under AgBSL-3 housing until euthanasia.

Experimental challenge was confirmed by two methods: recovery of the challenge strain, and seroconversion. Conjunctival swabs were taken at 5 days post-challenge, plated onto Kuzdas and Morse (KM) media [32], and incubated at 37 °C in 5% CO_2_ for 7 days to verify the presence of *B. melitensis* in each goat via colony morphology [33]. Isolates were confirmed as *Brucella* via polymerase chain reaction (PCR) using *Brucella*-specific primers for *omp2a* [34]. For serology, blood was obtained from the jugular vein at 0 and 4 weeks post-challenge in the short-term group and at 0, 2, 4, 8, 12, 23, and 37 weeks post-challenge in the long-term group. Antibody responses pre- and post-experimental challenge were evaluated using a standard tube agglutination test [33].

Large animal isolation facilities were operated under guidelines approved by the United States Department of Agriculture/Agricultural Research Service (USDA/ARS). All animal studies were performed under approval from the Institutional Animal Care and Use Committee (IACUC) at the NADC.

### Necropsy and tissue processing

Goats were euthanized by intravenous injection of sodium pentobarbital (Sleepaway, Ft. Dodge Labs, Ft. Dodge, IA, USA) at two different time points post-challenge. Samples obtained at necropsy for evaluation of bacterial content included: lymphatic tissues (parotid, prescapular (superficial cervical), retropharyngeal, and supramammary), placentome or uterus, and conjunctival swabs.

Tissue samples for bacterial enumeration were processed as previously reported [35, 36]. Briefly, approximately 1 g of each tissue sample was individually ground in 2 ml of PBS (pH = 7.2) using sterile glass Dounce homogenizers. Tenfold serial dilutions (10^−1^ to 10^−8^) of each suspension of each tissue were generated, and 100 μl of each dilution were plated onto KM and incubated at 37 °C in 5% CO_2_ for 7 days. The conjunctival swabs were streaked directly onto the surface of KM plates. All plates were incubated at 37 °C and 5% CO_2_ for 72 h, and isolates were identified as *Brucella* on the basis of colony morphology, growth characteristics, and a pan-*Brucella* PCR assay for the *omp2a* gene with the following primer set: 5′ TGG TCT GAA GTA TCA GGC TAC GCA 3′/5′ CCC AAG CAT TGT CTT CAG CAA CAG 3′.

### Isolation of *B. melitensis* 16M genomic DNA

Total bacterial genomic DNA was prepared from an overnight culture of *B. melitensis* 16M stock using the Ultra-Deep Microbiome Prep kit (Molzym Life Science, Germany) according to manufacturer’s recommendations.

### RNA isolation and cDNA preparation

Approximately 50–100 mg of each flash-frozen supramammary lymph node (SMLN) sample were pulverized with a pestle in a chilled mortar in liquid nitrogen and then immediately added to 1 ml of TRIzol reagent (ThermoFisher) and processed according to manufacturer’s instructions. The aqueous phase was collected and further purified using the Purelink RNA Mini kit (ThermoFisher), according to manufacturer’s recommendations. Residual DNA was removed from RNA samples by treating 1 μg of total RNA from each of the samples with 1 μl (2 U) of DNase I and 1 μl of 10× DNase I buffer (Life Technologies, Carlsbad, CA) in a total reaction volume of 11 μl. Samples were incubated at 37 °C for 30 min. for digestion and subsequently at 65 °C for 10 min. to inactivate the enzyme followed by the addition of 1.11 μl of 5 mM EDTA.

RNA quality was assessed with an Agilent Bioanalyzer, using the RNA 6000 Pico kit (Agilent, Santa Clara, CA). All extracted samples from SMLN (combination of host and pathogen RNA) had Bioanalyzer RIN scores of ≥ 7.7, with an average RIN score of 8.1 (Additional file 1: Table S1). RNA and DNA concentrations were measured using a NanoDrop (ThermoFisher).

cDNA was generated from total sample RNA using the method described in Azhikina et al. [28]. The first cDNA strand was synthesized using BR and SMART primers (Additional file 2: Table S2). Eleven μl of each DNase I-digested sample was mixed with 1 μl (10 pmol) BR and 1 μl (10 pmol) SMART primers, incubated at 70 °C for 2 min, and then held on ice for 10 min. For synthesis of first-strand cDNA, all 13 μl of annealed RNA were mixed with 0.5 μl of PrimeScript reverse transcriptase (RT) and the corresponding buffer (Clontech, Mountain View, CA) and incubated at 37 °C for 10 min, at 42 °C for 120 min, and at 95 °C for 5 min. Second-strand synthesis of cDNA was performed using 5S primer (Additional file 2: Table S2) and PrimeStar GXL polymerase and the corresponding buffer (Clontech, Mountain View, CA) for 30 cycles of 98 °C for 10 s, 64 °C for 20 s, and 68 °C for 5 min. Samples were then held at 68 °C for 10 min and then stored at 4 °C. The resulting cDNA was purified with a QIAquick PCR purification kit according to manufacturer’s recommendations (Qiagen, Valencia, CA).

To generate culture-derived RNA for coincidence cloning, total RNA from mid-log liquid cultures of strain *B. melitensis* 16M, cultured at 37 °C in BBL Brucella Broth (BD Biosciences, San Jose, CA), was isolated from cell pellets preserved with RNAProtect (ThermoFisher) using the PureLink RNA Mini Kit and subsequent DNase digestion, according to manufacturer’s recommendations.

### Coincidence cloning procedure

Coincidence cloning was performed as described by Azhikina et al. [28]. Separately, 1 μg of genomic DNA isolated from an overnight culture of *B. melitensis* 16M and 1 μg of cDNA from each lymph node sample were enzymatically digested with *Rsa*I (New England Biolabs, Ipswich, MA) for 4 h at 37 °C in the corresponding buffer, followed by *Rsa*I inactivation at 65 °C for 10 min. The fragments were ligated to specific suppression adapters: adapter I (T7NotSrf and Srf_10 mixture) with the genomic DNA, and adapter II (T7NotRsa and Rsa_10 mixture) with sample cDNA. Adapter-linked genomic DNA and adapter-linked sample cDNA were then mixed in hybridization buffer (50 mM HEPES, pH 8.3; 0.5 M NaCl; 0.02 mM EDTA, pH 8.0) and incubated at 99 °C for 5 min. (denaturation) followed by 68 °C for 18 h (renaturation).

The first PCR reaction (PCR1) was performed by combining 1 μl of the hybridized DNA-cDNA mixture with the external primer T7 (10 pmol; Additional file 2: Table S2) and PrimeStar GXL polymerase with corresponding PrimeStar Max buffer (Clontech, Mountain View, CA). The PCR1 reaction was carried out for 20 cycles of 98 °C for 10 s, 64 °C for 20 s, and 68 °C for 3.5 min, followed by a final hold step of 68 °C for 10 min. The second PCR reaction (PCR2) was performed using 1 μl of a 1:10 dilution of PCR1 product as a template, the internal primers Not1Srf (10 pmol) and Not1Rsa (10 pmol), and PrimeStar GXL polymerase with corresponding PrimeStar Max buffer (Clontech, Mountain View, CA) (Additional file 2: Table S2). The PCR2 reaction was performed using the same cycling conditions indicated for the PCR1 reaction. The product of the PCR2 reaction, or the “coincidence cloning product”, was purified using a QIAquick PCR Purification kit according to manufacturer’s recommendations (Qiagen, Valencia, CA).

### Library preparation and RNA-sequencing (RNA-Seq)

Amplicons were used for preparation of indexed libraries employing the Nextera XT DNA sample preparation and index kits according to manufacturer’s directions (Illumina, San Diego, CA). Resulting libraries were normalized, pooled and sequenced using the MiSeq v2 300 Cycle reagent kit, yielding 2 × 150-bp paired-end reads on the Illumina MiSeq platform (Illumina, San Diego, CA).

### Bioinformatic analysis

Paired-end sequencing reads for each sample were trimmed with Trimmomatic v. 0.35 [37]. Trimmed reads were aligned to a *B. melitensis* 16M reference genome (NCBI accessions NC_003317 and NC_003318, for chromosome I and II, respectively) with the bowtie2 v. 2.2.3 aligner [38]. HTSeq-count v. 0.6.1 was used to process the alignment files into gene-wise counts of reads mapped to the annotated genes in the reference genome [39]. SeqMonk v. 1.35 [40] was used to visualize the mapped reads and to perform basic quality control for the analysis. Cluster analyses were performed using R [41] and the following R packages: DESeq2 [42], gplots [43], ggplot2, PoiClaClu [44], RColorBrewer [45], magrittr [46], genefilter [47], and pheatmap [48]. The detection of insertions and deletions as compared to the *B. melitensis* 16M reference genome (NC_003317.1 and NC_003318.1) was performed using variant calling by FreeBayes [49] and SNVer (“SNVer for Individual Sequencing”) [50]. A filter requiring > 50 mapped reads at the indel location was applied. Indels presented in this manuscript were filtered for significance using a Bonferroni correction of p = 0.05/number of independent tests, and only significant reads are displayed in Additional file 6.

### Endpoint PCR reactions

To non-quantitatively detect the presence of *B. melitensis* 16M in cDNA samples, we utilized endpoint PCR reactions with Phusion DNA polymerase (New England Biolabs, Ipswich, MA), separated on 1% agarose gels with GelRed stain. The BMEI1305 primers (Additional file 2: Table S2) were used at a final concentration of 0.5 μM. Cycling conditions were as follows: 30 s at 98 °C denaturation step; 40 cycles of 10 s at 98 °C, 20 s at 64 °C, and 15 s at 72 °C; and 2 min of final elongation at 72 °C. The template for endpoint PCR reactions (25 μl total volume in 1× Phusion HF buffer) was double-stranded cDNA prepared from SMLN samples via the BR/SMART process as described above. The cDNA generated from RNA extracted from overnight *B. melitensis* 16M cultures, and processed in parallel to the lymph node samples, was used as a positive control. Negative controls, containing no added template, were run in parallel in all PCR experiments.

### qPCR and qRT-PCR assays

qPCR was utilized to determine the relative levels of transcription of a set of 5 *B. melitensis* 16M genes in long-term infection coincidence cloning samples. Primers were designed for each gene using NCBI’s PrimerSelect tool, and sequences are provided in Additional file 2: Table S2. The PCR1 product from the samples was used as a template in reactions performed with the SuperScript III Platinum SYBR Green One-Step qRT-PCR kit (ThermoFisher), following the manufacturer’s instructions (omitting the cDNA synthesis step). Briefly, reactions included 0.2 μM of each primer and 1 μl of a 1:10 dilution of PCR1 product, and were cycled on a Rotor-Gene Q (Qiagen, Valencia, CA) using the following program: 2 min at 95 °C; 40 cycles of 3 s at 95 °C and 30 s at 60 °C. For the comparison of *entA* and *ndvB*, a three-step cycling protocol was used for both primers to improve efficiency for the *ndvB* primer: 15 s at 95 °C, 30 s at 55 °C, and 30 s at 72 °C (also for 40 cycles). Amplification was detected using the SYBR Green signal. Separate endpoint PCR reactions were performed and the products were subsequently separated on an agarose gel to confirm the presence of single products for each primer set. Melt curve analysis was performed on each qPCR reaction to confirm the presence of a single peak per reaction. A template dilution series across a series of five concentrations (tenfold dilutions of template) was performed for each primer set to assess PCR efficiency. Average % efficiency values for the two-step cycling were as follows: *eryK* (erythritol kinase), 98%; *dksA*, 94%; *entA* (enterobactin), 99%; *ribE* (riboflavin synthase alpha subunit), 94%. For the comparison of *entA* and *ndvB* under three-step cycling conditions, the efficiency of *ndvB* was 82% vs. 92% efficiency for *entA*. We incorporated a correction factor to each C_t_ value based on the efficiency as compared to the efficiency of the *entA* primers.

C_t_ values were calculated using the Rotor-Gene Q software with manual determination of the threshold. C_t_ data for each gene were averaged across biological replicates. For some of the primer sets, amplification was observed in the no template control (presumably reflecting primer-dimer formation) before 40 cycles; however, C_t_ values for no template control reactions were > 10 cycles beyond C_t_ values for the experimental reactions in each case.

qRT-PCR was also attempted on short-term lymph node RNA samples using the gene-specific procedures described above. Prior to the cycling step, a 5 min hold step at 50 °C was utilized to convert RNA to cDNA via the amplification primers as gene-specific primers.

## Results

### Description of *B. melitensis* 16M infection in experimentally inoculated goats

**Fig. 1:**
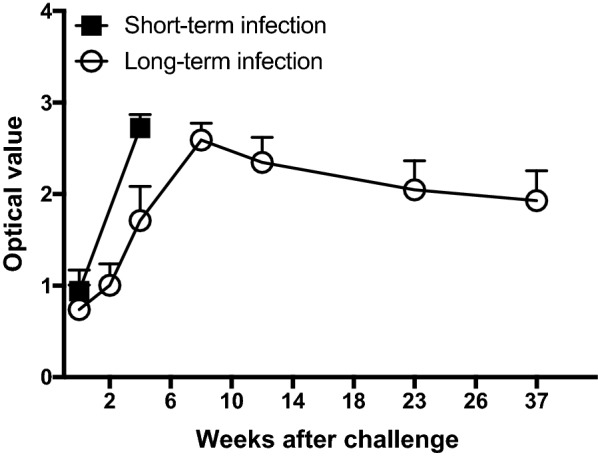
Anti-*Brucella* antibody production in response to *B. melitensis* 16M following infection. The standard tube agglutination test was used to measure anti-*Brucella* antibodies in serum collected at different time points post-infection for both the short-term (closed squares) and long-term (open circles) infection groups. Data points reflect the average of the optical values across each set of goats, ± the standard error of the mean

Goats were experimentally challenged with *B. melitensis* 16M for all experiments described below. Animals were placed into one of two groups: short-term infection (defined as 4 weeks post-challenge) or long-term infection (defined as 38 weeks post-challenge). The challenge strain was recovered from conjunctival swabs taken 5 days post-challenge, confirming successful infection. Both groups of animals demonstrated an increase in anti-*Brucella* serum antibodies against *B. melitensis* 16M (Fig. 1), additionally confirming infection. In the long-term infection group, antibody titers peaked at 8 weeks post-challenge and remained fairly stable with a minor decrease by the time animals were necropsied (Fig. 1).

### Bacterial tissue colonization in short- and long-term infection

Lymphoid (parotid, prescapular, and supramammary lymph nodes) and placentome/uterus samples were harvested during necropsy at 4 weeks (short-term infection) or at 38 weeks (long-term infection) post-challenge and were used to determine the number of *B. melitensis* 16M cells in the infected tissue. In the short-term infection group, *Brucella* were recovered from all tissues analyzed (Table 1), with the placentome containing the highest average log_10_ CFU/g (geometric mean; 6.95 ± 0.81). In contrast, we were unable to recover any viable *Brucella* from any of the tissues tested from the long-term infection group (Table 1).

**Table 1:** Animals utilized in the study, necropsy time points, and tissue colonization (expressed as log_10_ CFU/g)

	Animal no.	Breed	Pregnant (Y/N)	Fetus status	Infection date	Necropsy date	SMLN (log_10_ CFU/g)	Prescap (log_10_ CFU/g)	Parotid (log_10_ CFU/g)	Uterus/placentome (log_10_ CFU/g)
Short-term	1	Alpine	Y	Aborted	1/16/15	2/15/15	3.38	2.29	2.97	5.38
	2	Alpine	Y	Aborted	1/16/15	2/11/15	3.06	3.46	2.28	8.08
	3	Alpine	Y	Aborted	1/16/15	2/13/15	3.70	3.23	3.87	7.41
Long-term	4	Saanen	N	–	10/31/14	7/20/15	Neg	Neg	Neg	Neg
	5	Alpine	Y	Live birth	10/31/14	7/20/15	Neg	Neg	Neg	Neg
	6	Alpine	N	–	10/31/14	7/20/15	Neg	Neg	Neg	Neg
	7	Toggenburg	N	–	10/31/14	7/20/15	Neg	Neg	Neg	Neg

### Attempt to detect low levels of *B. melitensis* 16M in lymph nodes via existing nucleic acid methods

Recently, Wang et al. [51] demonstrated that low levels of *Brucella melitensis* could be identified more effectively via detection of RNA of highly expressed genes by RT-PCR, as compared to detection of genomic DNA by PCR, due to the presence of higher copy numbers of the RNAs. Wang et al. selected a *B. melitensis* gene for detection (BMEI1305, encoding the porin Omp2b) based on its absolute level of gene expression in a culture of *B. melitensis* grown to an OD_600_ of 1.0 at 37 °C in tryptic soy broth. The authors concluded that the BMEI1305 primer set is a candidate for ultrasensitive detection of the presence of *Brucella*. Since primers developed for BMEI1305 detection were designed based on gene expression levels in bacterial culture, and not an in vivo system, we wanted to determine if we could use this method to detect very low levels of bacteria in *B. melitensis* 16M-infected lymph nodes.

**Fig. 2:**
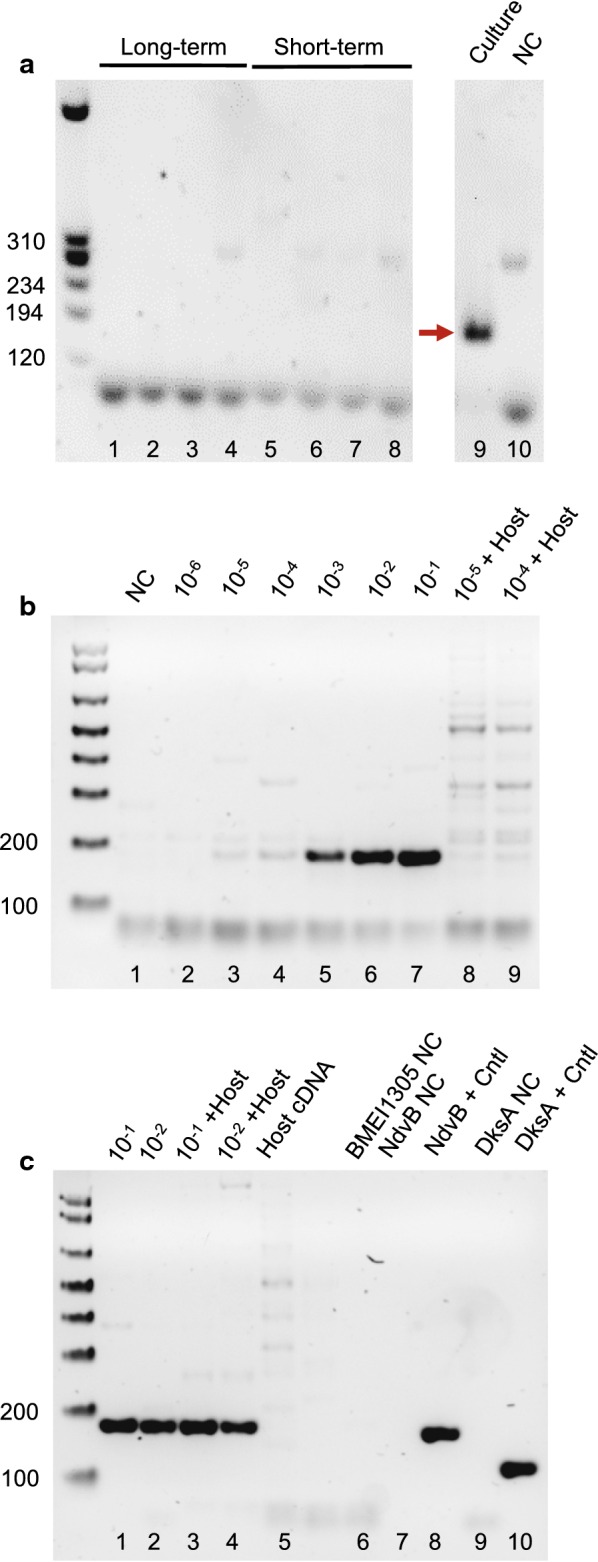
PCR methodologies with goat lymph node samples. **a** Attempt at detection of *Brucella* transcripts by endpoint RT-PCR. The BMEI1305 primers were used to amplify cDNA templates (0.5 µl each) derived from long-term samples (lanes 1–4), short-term samples (lanes 5–8; one sample reflected a short-term goat that was not included in the coincidence cloning profiling), or a *B. melitensis* culture cDNA sample as a positive control (lane 9), for 40 cycles. The culture cDNA sample was prepared in parallel to the lymph node cDNA samples, using equivalent starting amounts of total DNA; the red arrow indicates the amplified band of interest. NC = negative control with no template. Numbers presented to the left of each gel (**a**–**c**) are sizes of DNA ladder bands in base pairs. **b** Assessment of impacts of template dilution on amplification. As in (**a**), the BMEI1305 primers were used to amplify cDNA templates, in this case for 35 cycles. For lanes 2–7, 0.5 µl aliquots of serially-diluted *B. melitensis* culture-derived cDNA were added to each reaction; dilutions are indicated above each lane. In lanes 8 and 9, 0.5 µl of diluted bacterial cDNA was mixed with 0.5 µl of undiluted host cDNA in each reaction, with the bacterial dilution indicated above each lane. NC = negative control with no template. **c** Additional assessment of impacts of host cDNA template on PCR amplification, with reactions as described in (**b**) for 35 cycles. Dilution factors above each lane indicate the dilution factor of the culture-derived cDNA added to the reaction. Since multiple background bands were observed in the BMEI1305 reactions, we also completed negative control PCR reactions with the NdvB (lane 7) and DksA (lane 9) primers to demonstrate the absence of contamination in reactions. “+ control” reactions for NdvB (lane 8) and DksA (lane 10) contained 0.5 µl of culture-derived cDNA template

cDNA samples from the supramammary lymph nodes (SMLN) of each animal, prepared using the BR/SMART method, were used as templates for 40-cycle endpoint PCR reactions. cDNA prepared from an overnight *B. melitensis* 16M culture was used as a positive control, resulting in a band of approximately 180 base pairs (bp) in size, which is the expected product from the BMEI1305 primers (Fig. 2a, lane 9). However, with 0.5 µl of short-term or long-term lymph node cDNA added as template per reaction, the main product in each reaction was a primer-dimer band (Fig. 2a, lanes 1–8), as opposed to the main expected product. Dilution of the cDNA by up to 100× did not improve amplification (data not shown).

The absence of observable amplification could potentially be explained by the low levels of *Brucella* CFUs/g observed for the short-term lymph node tissues via culture methods (Table 1). Assuming an approximate value of 10^3^ CFUs/g, based on plated bacterial counts, homogenization of 100 mg of tissue for RNA processing would result in a theoretical maximum of only 100 CFUs per RNA sample, or ≈ 1 CFU/μl of RNA sample. This is at the limit of detection for the BMEI1305 primers that was reported for Wang et al. [51] and would require that extraction of pathogen RNA from the tissue samples be highly efficient. Alternatively, the highly abundant competing binding sites on the goat nucleic acid present in each sample may interfere with the PCR reactions. Assuming 500,000 mRNAs/eukaryotic cell, 5000 mRNAs/prokaryotic cell, and a mass of 1 × 10^−9^ g per mammalian cell, 1 g of lymph node tissue would contain ≈ 10^9^ host cells, as compared to 10^3^ bacterial cells, meaning that we could estimate the presence of 5 × 10^14^ host mRNAs in the gram of tissue vs. 5 × 10^6^ bacterial mRNAs. This estimated bacterial RNA: host RNA ratio (1:10^8^) is well below the expected bacterial RNA percentage (0.1–0.2%, or a 1:10^3^ ratio) that was estimated in the application of coincidence cloning by Azhikina et al. [28].

Therefore, in order to further assess the potential for nucleic acid detection using the BMEI1305 primers with lymph node samples, we characterized PCR reactions with increasing dilutions of *B. melitensis* cDNA derived from overnight bacterial cultures. The presence of host (goat) cDNA did not preclude amplification of *B. melitensis* cDNA when present at a ratio of 1:10^4^ pathogen cDNA:host cDNA (Fig. 2b, c). However, by a dilution of bacterial cDNA to 10^4^–10^5^, amplification of the expected band exhibited significant competition with off-target amplicons (Fig. 2b, lanes 2–7). We conclude that to characterize the nucleic acid inside *B. melitensis* 16M cells in lymph nodes, a more sensitive methodology with selective amplification of bacterial RNA is required.

### Detection and characterization of *Brucella melitensis* 16M via coincidence cloning

**Fig. 3:**
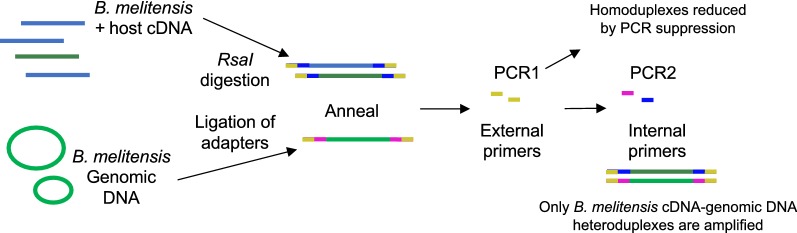
Overview of coincidence cloning procedure. Methodology is based on Azhikina et al. [28]. RNA extracted from SMLN tissues from each goat was used to generate total cDNA containing both host (blue) and bacterial (dark green) cDNA. Total cDNA and *B. melitensis* 16M total genomic DNA are fragmented using a restriction enzyme and the ends ligated with different suppressive adaptors (bacterial, host cDNA: yellow-blue; bacterial genomic cDNA: yellow-pink). Each cDNA sample is mixed with digested genomic DNA, and the mixture denatured and renatured in the presence of excess gDNA, generating both gDNA/gDNA homodimers and gDNA/cDNA heterodimers of complementary strands. The samples then undergo a two-step PCR amplification, resulting in selective enrichment of bacterial cDNA fragments. Libraries were prepared from amplified products and sequenced to obtain profiles of *B. melitensis* 16M gene expression within the tissue of infected hosts

We next examined whether coincidence cloning (Fig. 3) could be used for robust detection and characterization of the transcriptome of *B. melitensis* 16M in tissues from infected hosts. In selecting a tissue type for further processing, multiple sites of *Brucella* dissemination (as determined from the short-term culture data) were assessed for RNA quality. The quality of RNA from placental samples in short-term infected animals was not suitable for downstream RNA analysis, with RIN scores of < 6. However, RNA from lymph node samples was intact (Additional file 3: Table S3). As SMLN samples reflect a site of systemic dissemination of *Brucella* (based on intra-conjunctival infection), we selected RNA samples derived from these lymph nodes for further analysis.

For both the short-term and long-term infection groups, *Brucella* RNA was recovered from the samples, as indicated by sequencing reads mapping to the *Brucella melitensis* 16M genome. This indicates that even when bacterial cells are not recovered from lymph node via Dounce homogenization, *Brucella* cells can be present in the lymph node tissue. For example, bacterial cells may be retained internally in tissue samples post-homogenization, or retained internally inside intact macrophages or macrophage compartments. For each of the samples analyzed, approximately 1,000,000–1,500,000 read pairs were generated from sequencing, ≈ 75% of which mapped to annotated genes in the 16M genome in each case as determined by analysis by HTSeq (Table 2). This result indicates that the bacterial transcriptome was enriched evenly across samples, as compared to the genomic DNA used for coincidence cloning capture.

**Table 2:** Mapped reads per sample, generated from HTSeq-count mapping of reads to the *B. melitensis* 16M reference genome

Goat #	Total read pairs	Pairs in genes	Pairs not in genes	Pairs not mapping	% in genes
1	1,115,293	838,577	272,050	4666	75
2	1,400,935	1,067,233	328,057	5645	76
3	1,010,360	760,249	246,313	3798	75
4	1,544,084	1,199,374	338,847	5863	78
5	1,096,032	830,054	262,052	3926	76
6	968,576	720,841	244,936	2799	74
7	1,266,995	968,974	293,345	4676	76

**Fig. 4:**
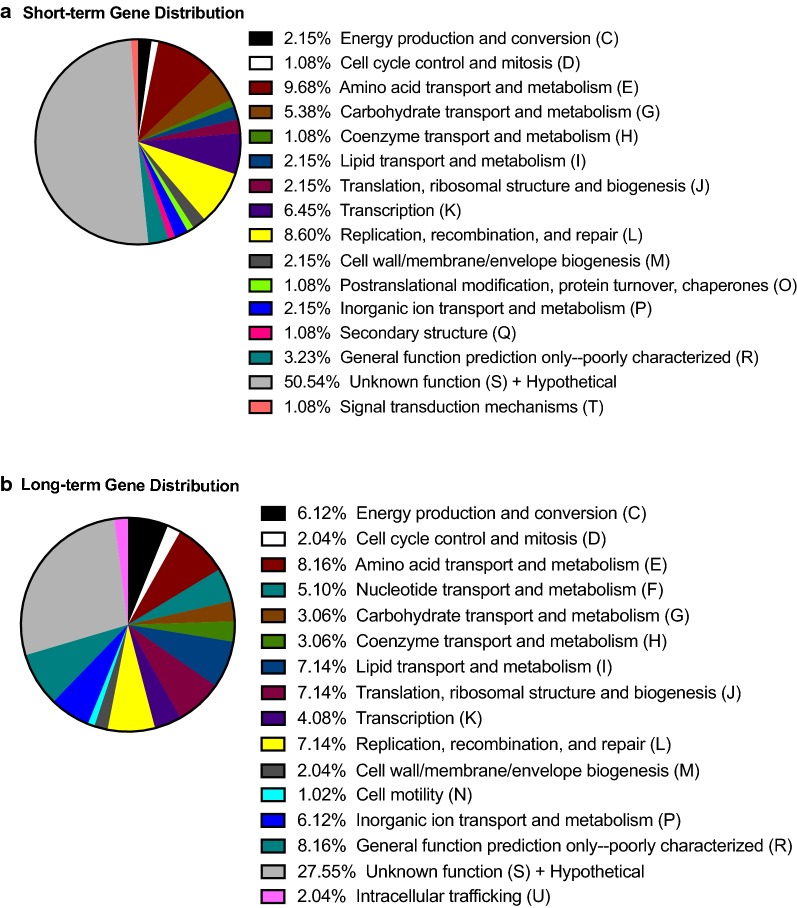
*B. melitensis* genes transcribed in SMLN from goats with short-term (**a**) or long-term (**b**) infections. For each sample set, the 100 genes with the highest levels of expression, based on normalization of counts to gene size as quantified by SeqMonk (FPKM), were categorized by COG category to generate the percentages in the pie charts. COG categories were retrieved from the PHIDIAS portal (www.phidias.us/bbp)

Next, we characterized the gene expression profile for each of the experimental groups. A full list of expression values for each *B. melitensis* 16M gene in short-term and in long-term infection is provided in Additional file 4. The top 100 genes ranked by expression from each of the experimental groups, based on counts normalized to gene and library size (FPKM), were categorized by functional annotation based on cluster of orthologous group categories (COG). The lists of annotated genes are included in Additional file 5, sorted by expression level for either the short-term or long-term samples. For the top 100 expressed genes in the short-term samples, we observed the following categories with the greatest representation: amino acid transport and metabolism (9.7%); replication, recombination, and repair (8.6%); and transcription (6.5%) (Fig. 4a). For the top 100 expressed genes in the long-term samples, we observed the highest percentage of genes in the categories of amino acid transport and metabolism (8.2%); lipid transport and metabolism (7.1%); translation, ribosomal structure and biogenesis (7.1%); and replication, recombination and repair (7.1%) (Fig. 4b). Therefore, the gene profile for both groups reflects a bacterial population that is metabolically active.

### Examining methodologies for verification of sample reads

When working with very low levels of *B. melitensis* cells per sample, environmental contamination, and/or sample cross-contamination, are a significant concern. Therefore, we examined two lines of evidence to determine whether the recovered RNA was from in vivo bacteria in the original lymph node samples, or due to contamination. The two lines of evidence considered were: (a) indel (insertion/deletion) analysis as compared to the *B. melitensis* 16M reference genome, and (b) clustering analysis of the samples, as compared to a set of comparison samples processed in parallel.

**Fig. 5:**
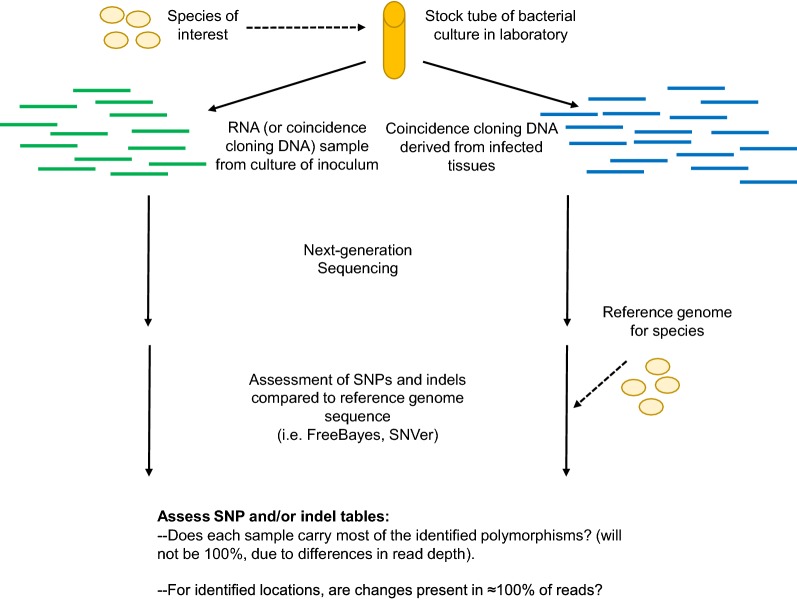
Schematic of work flow for indel assessment. Diagram depicts steps conducted in the paper to compare the recovered coincidence cloning reads to the original bacterial culture used for goat inoculation in this experiment. In this case, the species of interest was *B. melitensis*, with a culture of strain 16M from the National Animal Disease Center collection used for goat inoculation. The methodology exploits natural SNP variation between the stock tube culture and the published NCBI reference sequence for *B. melitensis* strain 16M for SNP (single nucleotide polymorphism) assessment. The procedure is presented as a suggested workflow for application of this methodology to future coincidence cloning experiments assessing samples with low pathogen abundance and, therefore, potential for environmental contamination

First, indel analysis (Fig. 5) was performed to identify insertions and deletions as compared to the *B. melitensis* 16M reference genome, in the seven SMLN-derived samples as well as a set of samples derived from coincidence cloning of *B. melitensis* RNA from bacterial cultures of the same master stocks in our laboratory. In coincidence cloning, only cDNA sequences matching the corresponding gDNA capture sequences should be recovered at the end of the procedure. Any sequence variations in the culture strain (used for both gDNA production and experimental inoculation) from the published reference should be observed in the resulting sequencing reads. Indeed, patterns for the tissue-derived samples were consistent with patterns for the reference culture samples. A table of indels across samples, generated by SNVer, is provided in Additional file 6. The majority of indels associated with the culture comparison samples were found in either all of the samples, including the culture controls (highlighted in green), or in all but one of the samples (highlighted in yellow); no indels were identified exclusively in all of the short-term or all of the long-term samples. Additional file 7 provides the annotation for multiple selected indel loci, presenting the percent of mapped reads carrying the sequence variation in representative samples. Importantly, the presence of gDNA in the heteroduplexes means that at least 50% of the reads should carry the sequence of the starting inoculum, so indel abundance of ≈ 100% at individual locations, paired with correspondence with the indels present in the culture data, is indicative of cDNA derived from the inoculated strain. These results indicate that the recovered RNA from the tissue-derived samples is consistent with the *B. melitensis* 16M cultures that were used to infect the animals. We suggest that the same methodology can alternatively be applied for future analysis using a simple RNA-sequencing run of RNA from the starting bacterial inoculum, as opposed to parallel coincidence cloning. This analysis is of particular value for laboratories processing numerous *Brucella* isolates.

**Fig. 6:**
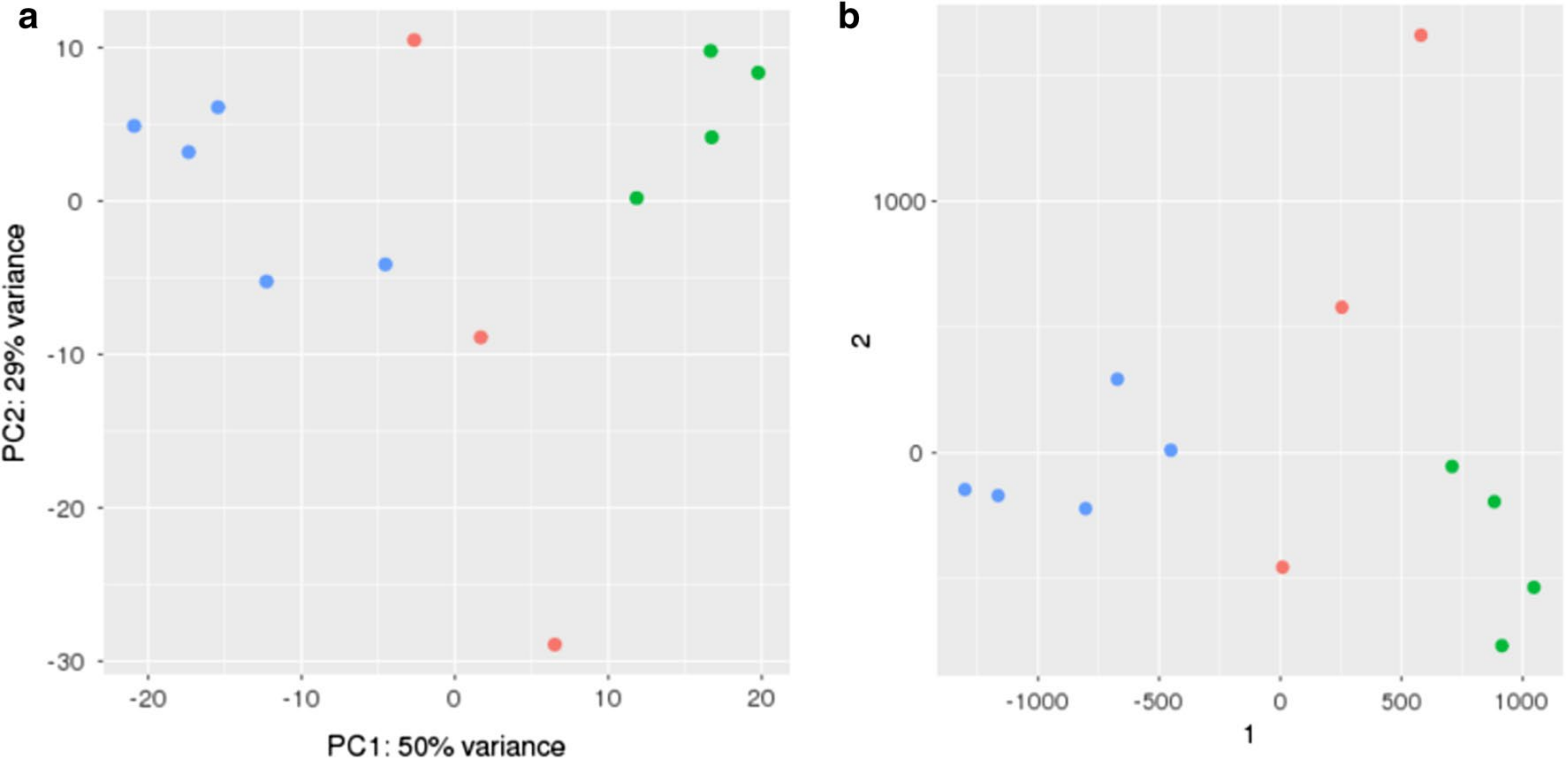
Clustering analysis of coincidence cloning samples. **a** Principal component analysis (PCA) plot and **b** multidimensional scaling (MDS) plot. The PCA plot used the overall sample distances generated from regularized log-transformation of the gene-wise count data (DESeq2). The MDS plots used the Poisson sample distances generated from the gene-wise count data (DESeq2). Green dots indicate long-term infection samples, red dots indicate short-term infection samples, and blue dots indicate culture samples for comparison

Second, we completed a clustering analysis to examine how the gene expression profiles for the short-term and long-term samples relate to each other, using the same set of culture-derived samples as a reference group for comparison. Via two means of clustering based on overall gene expression profiles, both the culture and the long-term infection samples each group together, whereas the short-term infection samples exhibit significant scatter (Fig. 6a, b). Despite the dispersion in the case of short-term samples, DESeq2 analysis of the short-term and long-term samples identifies a subset of differentially expressed genes (Additional file 8). The results suggest both that there are distinct profiles for culture and long-term infection samples, and that this methodology has the capacity to distinguish differential expression between groups.

### Validating gene expression patterns from coincidence cloning data

**Fig. 7:**
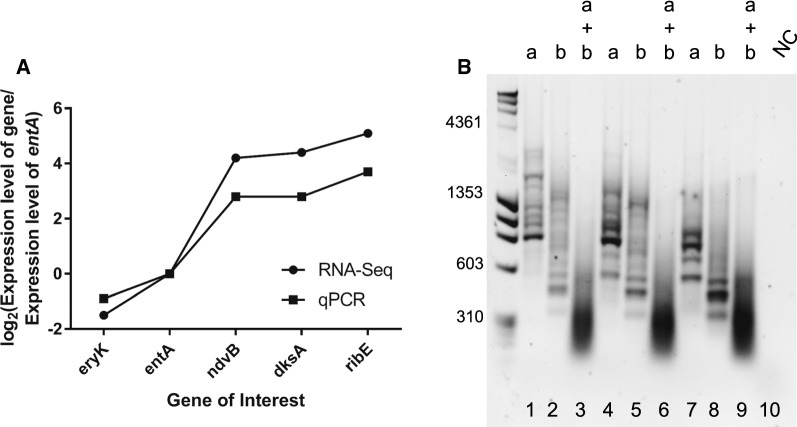
Assessment of the PCR1 products. **A** Assessment of gene expression levels in the PCR1 product. Actual vs. predicted gene expression levels of a set of five genes were assessed in the long-term infection samples via qPCR. PCR1 products from each long-term goat sample were amplified with five primer sets for the *ndvB*, *ribE*, *dksA*, *entA*, and *eryK* genes, as described in “Methods”. ΔC_t_ values were calculated for the expression of each gene relative to the expression level of the *entA* gene. The relative gene expression is expressed here as the log_2_ (expression level of gene of interest/expression level of *entA*), with closed squares for the qPCR-derived differences in expression and closed circles for the RNA-Seq-derived differences in expression (genes with higher expression than *entA* are depicted as positive values in this figure). Values were averaged across all four long-term goat samples (four biological replicates) to obtain the data points. **B** Assessment of suppression in the PCR1 reaction. PCR1 templates from three different tissue-derived samples (lanes 1–3, lanes 4–6, and lanes 7–9) were amplified under the PCR2 reaction parameters (described in “Methods”) with either Not1Srf primer (marked as “a”), Not1Rsa primer (marked as “b”), or both primers (marked as “a + b”). NC = negative control reaction, with no template but both primers. Numbers indicated on the left side of the gel indicate the molecular weight of ladder bands in base pairs

Next, we wanted to examine the feasibility of using qPCR to validate the profiles of relative gene expression levels generated from coincidence cloning. Therefore, we selected a series of 5 genes, with different levels of expression predicted from the analysis of the coincidence cloning data, and characterized their relative expression using PCR1 products from each of the long-term infected goat samples. The expression level of each gene was compared to the level of one of the other genes in the set (*entA*), and these results were compared with the relative levels of expression predicted from the (gene size-normalized) RNA-sequencing data set. The qPCR tests on the PCR1 products indicate good correspondence with the final sequencing results (Fig. 7A). This result suggests that the RNA-seq-characterized gene expression patterns are reflective of at least the PCR1 product.

Generation of the PCR1 product requires two sets of amplification, both in the cDNA preparation step and in the PCR1 amplification itself, which could lead to gene expression determinations that are not representative of the levels of each RNA in the original tissue. Additionally, the PCR1 product is generated after the addition of capture genomic DNA, which is a source of potential contamination. Therefore, we also attempted qRT-PCR reactions using RNA template from short-term infection samples with the *entA*, *ribE*, and *dksA* primer sets, but did not observe evidence of amplification beyond that in no template control samples (data not shown). Similarly, we did not observe amplification beyond that in no template control reactions for the primers with cDNA template from short-term infection samples. We also wondered whether the same level of “selective suppression of PCR” as observed previously for mycobacterium samples in the PCR1 reaction (repression of amplification of homoduplexes) was present for our *Brucella* samples. To test this, similar to the validation performed by Azhikina et al. [28], we completed a PCR2 amplification using single, internal primers (Not1Srf or Not1Rsa) on three PCR1 samples from infected goats. Notably, significant amplification was observed in the presence of only a single primer (Fig. 7B). This suggests that the PCR1 reaction did not suppress amplification of all of the genomic DNA that was mixed with the lymph node-derived sample. The larger size of the amplified products also suggests that we could be amplifying some larger genomic DNA or cDNA fragments that have not been digested completely with *Rsa*I. We conclude that there is at least some genomic DNA contamination in the PCR1 product, which would impact the assessment of relative gene expression levels via qPCR.

As a result, validation of gene expression patterns appears to be a challenge for the very low pathogen abundance samples in the *Brucella* coincidence cloning procedure. However, the appearance of the PCR2 reactions when both primers are used in the reaction (Fig. 7B, lanes 3, 6, 9) reaffirms that it is a separate set of products, presumably the desired cDNA-genomic DNA heteroduplexes, which are being amplified and highly enriched in the presence of both internal primers. Since *Rsa*I cuts at a 4 base pair sequence (GTAC), recognition sites are expected in the genome every 256 base pairs, consistent with the size of the PCR2 reactions with both primers. Therefore, the majority of the PCR2 products that are used for RNA-sequencing library preparation should reflect the transcripts that are present in the lymph node samples, and this is confirmed by the enrichment of reads mapping to bacterial genes in the RNA-sequencing samples. Additionally, > 99% of the reads aligned to the *B. melitensis* 16M genome (Table 2), so bacterial transcripts (vs. the eukaryotic host transcripts) have been exponentially enriched in the procedure. In contrast, only about 75% of the reads generated by pyrosequencing were bacterial in the original application of the procedure to mycobacterial infection by Azhikina et al. [28]; this result suggests that the PCR-based homoduplex suppression was not complete in this previous study as well.

### Identification of gene candidates of interest in long-term infection

Characterization of shifts in pathogen gene expression over time in the host provides the opportunity to identify genes and pathways important in pathogen persistence. First, we examined information provided by this dataset about the relative transcriptional profiles of *B. melitensis* bacteria under culture vs. long-term infection conditions. Our application of the coincidence cloning technique provided a profile of gene expression for *B. melitensis* in long-term culture (Additional files 4, 5) that was generated under conditions in which Brucella gDNA was present in vast excess to cDNA, as desired for effective and unbiased hybridization. However, in culture-derived samples such as the ones depicted in Fig. 6, the same excess of gDNA is not present. Therefore, to avoid impacts of hybridization bias, we compared the gene expression profile from *Brucella* recovered after 38 weeks of infection (Additional file 4) to a previously published dataset of gene RPKM values for *B. melitensis* 16M grown at 37 °C in acidified culture [52], as an in vitro mimic of macrophage conditions in the absence of other adaptations to the host environment.

We identified a subset of genes that were among the top in expression (normalized to gene length) in our dataset, and simultaneously near the bottom of the list of expression for the acidified in vitro dataset from Liu et al. [52]. Next, we further narrowed genes within that subset to those that were also upregulated > 4-fold in our long-term lymph node vs. in vitro (nonacidified) culture coincidence cloning dataset. Resulting candidate genes of interest are presented in Table 3. The majority of the selected genes were not significantly influenced by growth phase in culture, based on data from Rossetti et al. [53], suggesting that the observed differences are not simply a function of the growth phase of the culture sample selected for comparison. These genes serve as candidates for future study of their roles in the persistence of *Brucella* in the host. Of particular interest is the phosphatidate cytidylyltransferase gene (*cdsA*), which is involved in synthesis of phospholipid precursors from sn-glycerol-3-phosphate (the transporters of which are also candidates of interest; Table 3). Indeed, the long-term samples exhibit high levels of expression of genes involved in lipid metabolism (Fig. 4b).

**Table 3:** Candidate genes of interest based on abundance in long-term *Brucella* samples

Locus tag	Product name/description	Rank in acidified culture [52]	Rank in long-term	Log_2_Expn in LT vs. culture	Growth phase reg. [53]	Reg. in cattle (4 h) [27]	Reg. in MΦ (24 h) [54]
BMEI0050	CobT (Cobalamin biosynthesis)	2043	41	2.7	N	Mixed effects	No change
BMEI0460	MoxR family protein	1892	42	3.1	N	Repressed	Slight repress
BMEI0461	Hypothetical protein	1971	31	3.1	N	No change	Slight repress
BMEI0828	Phosphatidate cytidyltransferase	2101	95	5.9	N	Activated	Slight repress
BMEI1817	ATP-dependent helicase HrpB	3100	1291	6.6	N	Repressed	No change
BMEI1896	Hypothetical protein	2953	17	2.8	N	Repressed	No change
BMEII0111	ICC protein family	2227	136	3.2	N	Activated	No change
BMEII0113	sn-Glycerol-3-phosphate transport system UgpA	3026	94	3.2	N	Repressed	No change
BMEII0114	sn-Glycerol-3-phosphate transport system UgpE	2611	254	2.9	Y	Repressed	No change
BMEII0167	FlhA2 flagellar biosynthesis protein	2902	71	2.9	N	Repressed	No change
BMEII0831	Hypothetical protein	1937	253	7.7	N	Activated	No change
BMEII0832	UDP-glucose-4-epimerase	3128	239	7.5	N	Repressed	No change

Relevant expression information for each gene candidate is compiled from external data sources as described, plus the coincidence cloning data set presented in this paper. Rank in acidified culture: Rank of gene in Liu et al. [52] expression set, sorted from high (1) to low (3152) by RPKM. Rank in long-term: Rank of gene in averaged long-term expression set from coincidence cloning (CC), sorted from high (1) to low (3264) by gene expression normalized to gene and library size in SeqMonk (FPKM). Log_2_Expn: log_2_ (expression in long-term samples/expression in culture-derived samples) for CC dataset, generated as log_2_ (FPM for long-term/FPM for culture) in SeqMonk. Growth phase reg: Expresses whether (yes/no) the gene locus was identified as exhibiting a significant and > 2-fold change in gene expression between log and stationary phase in culture by Rossetti et al. [53]. Reg. in cattle: describes pattern of gene expression observed over the first 4 h. of introduction of cultured *B. melitensis* to cattle intestinal sections [27]. Reg. in macrophages: Describes pattern of gene expression observed 24 h post-murine macrophage infection, as compared to expression pre-infection in broth culture [54]. Data from Liu et al. [52] reflect actively growing cultures in vitro in Tryptic Soy Broth (fresh media added after subculture from stationary phase), exposed to acid stress like that expected in a macrophage (pH 3.4). Data from the culture-derived coincidence cloning (CC) samples was obtained from parallel CC of RNA derived from overnight cultures of the *B. melitensis* 16M challenge strain grown in Brucella broth

Additionally, we examined the differential gene expression profiles of the short-term and long-term *Brucella* samples, as outlined in Additional file 8, for genes of interest for *B. melitensis* persistence in vivo. Specifically, we selected genes from Additional file 8 with (a) a p-value of < 0.01 for significance (in difference in expression between the short-term and long-term groups) and (b) an increase in expression of > 8-fold from the short-term to the long-term condition. In other words, these are genes that exhibit significant upregulation between 4 and 38 weeks post-challenge in the goat (Table 4). We then compared this list of genes to a list of *Brucella* virulence factors, based on their mutants’ attenuation in mice, macrophages, or HeLa cells, as compiled from the literature by He [55] and Brambila-Tapia et al. [56] (basis for far right column, Table 4). Notably, three of the highly upregulated genes are known virulence genes: BMEI1766 (*cysI*), BMEII0527 (*xseA*), and BMEII0089 (*rbsK*). Other genes in Table 4 may be (a) upregulated in persistent infection but not essential for virulence, or (b) essential for persistence in the long-term goat model, but not essential for virulence in shorter-term models of infection in mice or cells. Indeed, significant disparity is expected between lists of genes required for virulence in short-term infection, and genes that are distinctly upregulated during long-term infection.

**Table 4:** Candidate genes of interest in persistence based on upregulation in long-term (vs. short-term) samples

Locus tag	Product name/description	Log_2_ (fold change)	Listed as virulence gene?
BMEII1062	(S)-2-hydroxy-acid oxidase subunit GlcE	4.8	No
BMEII1029	3-Deoxy-d-manno-octulosonic-acid transferase	4.3	No
BMEI1927	Enoyl-CoA hydratase	4.1	No
BMEII0089	Ribokinase (*rbsK*)	4.1	Yes
BMEII0086	Galactoside transport system permease MglC	4.0	No
BMEI1816	Sensory transduction protein kinase	4.0	No
BMEII0524	Hypothetical protein	3.7	No
BMEII0860	Oligopeptide transport system permease protein AppB	3.7	No
BMEII0862	Dihydrodipicolinate synthase	3.6	No
BMEI0234	Hypothetical cytosolic protein	3.5	No
BMEI1766	Sulfite reductase (ferredoxin; *cysI*)	3.4	Yes
BMEII0527	Exodeoxyribonuclease VII large subunit (*xseA*)	3.4	Yes
BMEII0526	Transcriptional regulatory protein, LysR family	3.4	No
BMEI0171	Ribosomal protein L11 methyltransferase	3.4	No
BMEI1926	Hydroxymethylglutaryl-CoA lyase	3.4	No
BMEI1817	ATP_dependent helicase HrpB	3.3	No
BMEI0173	Ycil-like protein	3.3	No
BMEI1925	Acetyl-CoA carboxylase alpha chain/propionyl-CoA carboxylase alpha chain	3.3	No
BMEII0564	Bifunctional proline dehydrogenase/pyrroline-5-carboxylate dehydrogenase (*putA*)	3.2	No
BMEII0079	Isochorismatase	3.2	No
BMEI0248	F0F1 ATP synthase subunit delta	3.1	No
BMEII0864	Oligopeptide transport ATP-binding protein AppF	3.1	No
BMEII1030	Putative lipoprotein	3.0	No
BMEII0084	Basic membrane protein A precursor	3.0	No
BMEII0083	Basic membrane protein A precursor	3.0	No

Genes are listed in the order of fold-effect, with the fold change reflecting an increase in expression in the long-term samples in each case (see Additional file 8 for details on differential gene expression analysis; log_2_ (fold effect) values are displayed based on the analysis in Additional file 8). Gene descriptions are compiled from the Brucella Bioinformatics Portal (www.phidias.us)

Other genes of note in Table 4 include BMEII1029, BMEII0564, and BMEI1816. BMEII1029 encodes 3-deoxy-d-manno-octulosonic-acid transferase; this gene has been demonstrated to contribute to synthesis of lipooligosaccharide for the outer membrane of *Moraxella catarrhalis*, and was important in virulence in a mouse model [57]. A recent paper on *B. abortus* demonstrated that the homolog of PutA (BMEII0564 in *B. melitensis*), an l-proline dehydrogenase, was important in the replication and survival of the bacterium in the murine host [58]. Finally, the protein product encoded by BMEI1816 is a homolog to the RegB sensor histidine kinase in *B. suis*; the RegA/RegB system in *B. suis* is involved in adaptation to oxygen depletion, and is required for murine chronic infection [59]. Recent work suggests that the RegB/A system is critical for adaptation to the conditions of chronic infection in the host, in response to detection of low oxygen levels [60].

## Discussion

In this paper, we provide the first application of the coincidence cloning technique to the analysis of *B. melitensis* strain 16M from an infected animal host. This is also the first characterization of *Brucella* RNA from bacterial cells that have disseminated in infection to lymph nodes. We demonstrate the ability to recover sufficient *Brucella* RNA from infected host lymph nodes for the coincidence cloning technique. Coincidence cloning enabled us to obtain sequencing reads sufficient to characterize in vivo *Brucella* gene expression across the genome, despite the extremely low levels of starting material. Additionally, we developed a rapid workflow, using the analysis of indels, to assess the identity of the reads recovered by coincidence cloning as the input strain.

The work presented here utilized two groups of *B. melitensis* 16M-infected goats: a short-term group (4 weeks post-challenge) and a long-term group (38 weeks post-challenge). In the short-term group, bacterial colonization of infected tissues ranged from 10^3^ to 10^8^ CFU/g, consistent with our previous findings [61, 62]. Interestingly, none of the tissues analyzed in the long-term group yielded culturable bacteria, using the homogenization methods described here. However, these tissues tested positive for *B. melitensis* 16M via the coincidence cloning technique. This raises two possible scenarios: either the culture techniques utilized in this paper are not sensitive enough at this stage of infection, or the recovered nucleic acid reflects low levels of background contamination. To determine bacterial load in this study, 1 g of whole tissue was homogenized in 2 ml of PBS, and serial dilutions of this homogenate were plated. The use of glass (Dounce) homogenizers to grind tissue typically preserves organelle structure, so the inability to recover *Brucella* from the long-term tissue could indicate difficulties in recovering bacteria from their location in vesicles inside of macrophages. In contrast, the TRIzol lysis buffer combined with mechanical homogenization in our RNA extraction protocol would be able to recover nucleic acid from the intracellular (and intravesicular) environment. In future work, we would consider performing enrichment steps, such as selective lysing of host cells or preincubation in enrichment media prior to plating, to increase bacterial yield.

While background contamination is a reasonable concern, multiple lines of evidence indicate that the isolated nucleic acids did not originate from environmental contaminants. First, potential contaminating nucleic acids would more likely be stable genomic DNA, which would be removed or at least highly reduced during the DNase treatment step. The PCR amplification steps (PCR1 and 2) in the coincidence cloning procedure also limit amplification to linker-attached cDNAs; therefore, any potential contaminants introduced during later stages of the procedure would not be amplified. In addition, the RNA-Seq reads correspond with the sequence of the *B. melitensis* 16M isolate that was used to challenge the animals, as determined by indel analysis of the transcriptome (Additional file 6). Finally, we observe a distinct profile for the long-term infection samples when gene expression profiles are analyzed by principal component and multidimensional scaling analyses (Fig. 6). While we cannot completely exclude the possibility that the profiles result from contaminating RNA in the samples, the evidence presented above supports the conclusion that we are observing nucleic acid isolated and amplified from the tissue samples, despite the lack of culturable bacteria.

Recently, the use of dual RNA-seq methods, in which combined samples can be analyzed by deep sequencing to assess both host and pathogen gene expression profiles, has expanded to assess multiple types of pathogens [63]. In some of these cases, selective depletion of host transcripts was utilized. While the majority of this work has focused on in vitro infection of cells, in the past few years, analysis has expanded to include in vivo infections in the case of certain pathogens (for example, *Toxoplasma gondii* [64], *Yersinia pseudotuberculosis* [65] and *Pseudomonas aeruginosa* [66]). In the case of *Brucella* infection, Rossetti et al. characterized changes in bacterial and pathogen transcriptomes, but this was performed over the course of a 4 h time period subsequent to introduction of 3 × 10^9^ CFU of bacteria [27]. The very low levels of *Brucella* present during long-term infection present additional challenges for sample analysis. With the use of coincidence cloning, we were able to obtain over 1 million *Brucella*-specific read pairs from very low abundance samples, and were also able to select for *Brucella* transcripts as opposed to transcripts from other potentially contaminating bacteria. Thus, the coincidence cloning procedure may have a niche in terms of usage for very low abundance mixed samples. Future work comparing sensitivity and gene expression patterns between the coincidence cloning method and the selective amplification method described by Rossetti et al. [29] would also be useful in advancing *Brucella* transcriptome profiling.

However, the inability to validate gene expression patterns directly from RNA or cDNA samples in cases with extremely low ratios of bacterial:host RNA makes it difficult to assess the impact of potential biases introduced during the annealing and amplification steps of the coincidence cloning procedure. Therefore, we propose that the best option for future studies with *Brucella* is to collect a series of samples across the time course of infection, as opposed to comparisons to the gene expression in bacterial pure cultures. Then, impacts of gene recovery bias will be less critical, since relative changes in expression across the infection time course will be the basis for identification of genes of interest in bacterial virulence. In the comparison of short-term and long-term gene expression profiles conducted here, as a first look at potential genes of interest for persistence, genes that are upregulated in long-term vs. short-term infection are potential candidates for deletion in brucellosis vaccine development, as their deletion has the potential to disrupt establishment of persistent infection without compromising early development of a protective response against the vaccine strain.

## Conclusions

The majority of transcriptomic experiments in *B. melitensis* have examined changes in gene expression in the initial stages of infection. However, *Brucella* sets up a persistent infection in many cases, with host immune responses occurring over time frames of weeks as opposed to days. Therefore, an understanding of pathogen gene expression changes over the long term is important in our understanding of persistent infections.

Here, we demonstrate the application of the coincidence cloning technique to long-term *Brucella* infections, describe transcriptional profiles of bacteria in lymph nodes from both long-term and shorter-term infections, and discuss challenges and potential solutions related to the analysis of samples with very low pathogen nucleic acid abundance. We also identify gene candidates of interest exhibiting unexpectedly high transcriptional abundance in long-term samples, including genes involved in lipid metabolism, or exhibiting significant upregulation of expression in long-term infection as compared to short-term infection, such as proline utilization and cellular signaling (bacterial two-component systems) genes that have been recently implicated in other model systems for *Brucella* infection. These gene candidates will serve as starting points for characterization of changing RNA profiles over time in different *Brucella* species. Future extension of this study to capture a time course of samples from *Brucella*-infected animals may allow for identification of distinct stages of infection via transcriptional profiling. This type of information would be highly informative in understanding how *Brucella* spp. adapt to their intracellular environment in the mammalian host, and in identifying which genes are critical for their maintenance and survival. Characterization of such genes could identify important targets for therapeutic intervention, as well as for modification in vaccine development to reduce host persistence of attenuated strains.

## Additional files

**Additional file 1: Table S1.** RIN scores for extracted supramammary lymph node (SMLN) samples: table includes information regarding goat number, experimental group and the RIN score for each SMLN extracted and used in the study.

**Additional file 2: Table S2.** List of primers used in all experiments: table includes a full list of all primers, and their respective sequences, utilized in the study.

**Additional file 3: Table S3.** RNA quality and yields from *B. melitensis*-infected goat samples.

**Additional file 4.** Gene expression values for short- and long-term goat samples. Annotated probe table prepared for coincidence cloning samples for goats 1–3 (short-term) and goats 4–7 (long-term) using SeqMonk. Libraries were treated as paired-end upon loading into SeqMonk. Quantitation was performed using the RNA-Sequencing pipeline in SeqMonk, using the option for normalization of values to gene size (per kilobase of transcript per million mapped reads, or FPKM, based on SeqMonk documentation), and expression values are expressed as log_2_ transformations of FPKM values. Start and end columns indicate locations of each quantitated feature in *the B. melitensis* 16M reference genome.

**Additional file 5.** Sorted gene expression tables of top 100 genes by relative expression. Average gene length-normalized expression values (FPKM, calculated by SeqMonk) for the short-term and long-term coincidence cloning samples, generated as described in Additional file 4, were sorted by expression level for the short-term (left-hand columns) or the long-term (right-hand columns) samples. COG (Cluster of Orthologous Group) designations, as determined via the PHIDIAS Brucella Bioinformatics Portal (www.phidias.us/bbp/), are annotated. The top 100 genes from each group in expression levels are provided. We note that there are challenges with the use of read data in FPKM/RPKM averaged across multiple samples, as discussed by Wagner et al. [67]. However, as depicted in Additional file 4, average FPKM values for each sample were very similar between biological replicates. We also completed an analysis in which the rank orders of gene expression were determined for each individual sample, and then genes were sorted by the lowest average rank order of expression. 96% of the long-term top 100 genes and 90% of the short-term top 100 genes were identical between the two methods. COG category single letter associations: amino acid transport and metabolism (E), carbohydrate transport and metabolism (G), cell cycle control and cell division, chromosome partitioning (D), cell motility (N), cell wall/membrane/envelope biogenesis (M), chromatin structure and dynamics (B), coenzyme transport and metabolism (H), cytoskeleton (Z), defense mechanisms (V), energy production and conversion (C), extracellular structures (W), function unknown (S), general function and prediction only (R), inorganic ion transport and metabolism (P), intracellular trafficking, secretion and vesicular transport (U), lipid transport and metabolism (I), nucleotide transport and metabolism (F), post-translational modification, protein turnover, chaperones (O), RNA processing and modification (A), replication, recombination and repair (L), secondary metabolites biosynthesis, transport and catabolism (Q), signal transduction mechanisms (T), transcription (K), translation, ribosomal structure and biogenesis (J).

**Additional file 6.** Indel analysis table across coincidence cloning samples. This table of indels present in the coincidence cloning reads was generated by SNVer analysis, as described in Methods, with only indels passing the read depth and significance filters displayed here. Columns A and B indicate the reference location of each indel on the chromosome (either I, indicated by NC_003317.1, or II, indicated by NC_003318.1). A “0” indicates the absence of the indel as compared to the *B. melitensis* 16M reference genome (matching the reference sequence) and a “1” indicates the presence of the indel. “Cultures 1–5” are *B. melitensis* broth culture-derived coincidence cloning samples, and the remaining samples are listed by their goat number. Rows in which all samples carried the designated indel are colored in green, and rows in which all but one of the samples carried the designated indel are colored in yellow. Please note that in cases where some samples did not exhibit the indel, this can be due to a read depth below the cutoff of > 50 at the indel location for that sample, as used for the SNVer filter.

**Additional file 7.** Additional indel analysis. Data is presented from FreeBayes for a set of 5 selected indels identified by both FreeBayes and SNVer for coincidence cloning reads as compared to the *B. melitensis* 16M reference genome. For each of the samples indicated (provided as examples), the following are displayed: the total reads at the indel location from the RNA-sequencing sample, as mapped to the *B. melitensis* genome; the number of reads carrying the alternative (indel) allele; and the percent of mapped reads exhibiting the indel. > 92% of reads in each displayed location carried the indel of interest.

**Additional file 8.** Analysis of differential gene expression comparison between short-term and long-term samples. Table presents the results of DESeq2 differential expression analysis comparing the group of short-term samples (goats 1–3) and long-term samples (goats 4–7). Data are sorted by the adjusted p-value for each comparison (Column G), from smallest to largest. Genes are identified by the locus tag in Column A. The log_2_FoldChange (Column C) indicates the log_2_ of the differential gene expression of (Long-term/Short-term) samples.

## Abbreviations

CC: coincidence cloning
SMLN: supramammary lymph node
