# Supplementary material for: Coincidence cloning recovery of Brucella melitensis RNA from goat tissues: advancing the in vivo analysis of pathogen gene expression in brucellosis
Source: BMC Mol Biol. 2018 Aug 1;19:10. doi: 10.1186/s12867-018-0111-x (PMC6071331; doi:10.1186/s12867-018-0111-x)
Supplement: Supplementary file 1 — Additional file 1: Table S1. RIN scores for extracted supramammary lymph node (SMLN) samples: table includes information regarding goat number, experimental group and the RIN score for each SMLN extracted and used in the study. [file 12867_2018_111_MOESM1_ESM.docx]

**Table S1.** RIN scores for extracted supramammary lymph node (SMLN) samples.

| **Goat No.** | **Group** | **RIN Score** |
| --- | --- | --- |
| 1 | Short-term | 8.2 |
| 2 | Short-term | 8.0 |
| 3 | Short-term | 8.6 |
| 4 | Long-term | 7.9 |
| 5 | Long-term | 8 |
| 6 | Long-term | 7.7 |
| 7 | Long-term | 8.2 |
