# Supplementary material for: Coincidence cloning recovery of Brucella melitensis RNA from goat tissues: advancing the in vivo analysis of pathogen gene expression in brucellosis
Source: BMC Mol Biol. 2018 Aug 1;19:10. doi: 10.1186/s12867-018-0111-x (PMC6071331; doi:10.1186/s12867-018-0111-x)
Supplement: Supplementary file 2 — Additional file 2: Table S2. List of primers used in all experiments: table includes a full list of all primers, and their respective sequences, utilized in the study. [file 12867_2018_111_MOESM2_ESM.docx]

**Table S2. List of primers used in all experiments.**

| **Primer Name** | **Amplified Gene** | **Sequence** | **Source** |
| --- | --- | --- | --- |
| **BR** | N/A | 5’ AAGCAGTGGTATCAACGCAGAGTAC(N)_9_ 3’ | Azhikina et al., 2010 |
| **SMART** | N/A | 5’ AAGCAGTGGTATCAACGCAGAGTACGCrGrGrG 3’ | Azhikina et al., 2010 |
| **5S** | N/A | 5’ GTGGTATCAACGCAGAGT 3’ | Azhikina et al., 2010 |
| **T7 NotSrf** | N/A | 5’ CTAATACGACTCACTATAGGGCTCGAGCGGCCGCCCGGGC AGGT 3’ | Azhikina et al., 2010 |
| **Srf_10** | N/A | 5’ ACCTGCCCGG | Azhikina et al., 2010 |
| **T7NotRsa** | N/A | 5’ CTAATACGACTCACTATAGGGCAGCGTGGTCGCGGCCG AGGT 3’ | Azhikina et al., 2010 |
| **Rsa_10** | N/A | 5’ ACCTCGGCCG 3’ | Azhikina et al., 2010 |
| **Not1Srf** | N/A | 5’ TCGAGCGGCCGCCCGGGCAGGT 3’ | Azhikina et al., 2010 |
| **Not1Rsa** | N/A | 5’ AGCGTGGTCGCGGCCGAGGT 3’ | Azhikina et al., 2010 |
| **T7** | N/A | 5’ CTAATACGACTCACTATAGGGC 3’ | Azhikina et al., 2010 |
| ***dksA* F** | BME_RS04720  (BMEI0949) | 5’ ATGCTGCATTGAGCCGTTTG 3’ | This paper |
| ***dksA* R** | BME_RS04720  (BMEI0949) | 5’ GAAAGTGTTGCGATCGGACG 3’ | This paper |
| ***eryK* F** | BME_RS12295  (BMEII0430) | 5’ CACAATCGCTTGCCCAAACA 3’ | This paper |
| ***eryK* R** | BME_RS12295  (BMEII0430) | 5’ GATCCAGGCATCACCTACCG 3’ | This paper |
| ***entA* F** | BME_RS10575  (BMEII0079) | 5’ CGAGGATGAGACGGAACTCG 3’ | This paper |
| ***entA* R** | BME_RS10575  (BMEII0079) | 5’ CGCGGGCAAGATCATCAAAG 3’ | This paper |
| ***ribE* F** | BME_RS05980  (BMEI1188) | 5’ GTATGCCTCACGGTTGTTGC 3’ | This paper |
| ***ribE* R** | BME_RS05980  (BMEI1188) | 5’ ACCACCCATTTCATCGCCAA 3’ | This paper |
| **BMEI1305-RT-F** | BME_RS06565  (BMEI1305) | 5’ TACCTTCACCGGTTACCTCG 3’ | Wang et al., 2016 |
| **BMEI1305-RT-R** | BME_RS06565  (BMEI1305) | 5’ CGATGTGGTAGTTGGTCGTG 3’ | Wang et al., 2016 |
| ***ndvB* F** | BME_RS09095 (BMEI1837) | 5’ GCGTTCAGGTGAAAACACCC 3’ | This paper |
| ***ndvB* R** | BME_RS09095 (BMEI1837) | 5’ GCGTTCAGGTGAAAACACCC 3’ | This paper |
