# Supplementary material for: Coincidence cloning recovery of Brucella melitensis RNA from goat tissues: advancing the in vivo analysis of pathogen gene expression in brucellosis
Source: BMC Mol Biol. 2018 Aug 1;19:10. doi: 10.1186/s12867-018-0111-x (PMC6071331; doi:10.1186/s12867-018-0111-x)
Supplement: Supplementary file 3 — Additional file 3: Table S3. RNA quality and yields from B. melitensis-infected goat samples. [file 12867_2018_111_MOESM3_ESM.docx]

**Table S3.** RNA quality and yields from *B. melitensis*-infected goat samples.

| **Tissue Type** | **Average RIN Score** | **Approximate Yield (μg)**  **(from 50-100 mg tissue pieces)** |
| --- | --- | --- |
| Supramammary Lymph Node | 8.1 | 220 |
| Parotid Lymph Node | 8.1 | 180 |
| Prescapular Lymph Node | 8.0 | 250 |
| Placentome-related tissue* | 5.7 | 180 |
